# Carbon Nanofibers versus Silver Nanoparticles: Time-Dependent Cytotoxicity, Proliferation, and Gene Expression

**DOI:** 10.3390/biomedicines9091155

**Published:** 2021-09-03

**Authors:** Beatriz Salesa, Marcelo Assis, Juan Andrés, Ángel Serrano-Aroca

**Affiliations:** 1 Biomaterials and Bioengineering Lab., Centro de Investigación Traslacional San Alberto Magno, Universidad Católica de Valencia San Vicente Mártir, c/Guillem de Castro 94, 46001 Valencia, Spain; beatriz.salesa@ucv.es; 2 Department of Physical and Analytical Chemistry, University Jaume I (UJI), 12071 Castellon, Spain; marcelostassis@gmail.com (M.A.); andres@qfa.uji.es (J.A.)

**Keywords:** silver nanoparticles, carbon nanofibers, human keratinocytes, cytotoxicity, proliferation, gene expression

## Abstract

Carbon nanofibers (CNFs) are one-dimensional nanomaterials with excellent physical and broad-spectrum antimicrobial properties characterized by a low risk of antimicrobial resistance. Silver nanoparticles (AgNPs) are antimicrobial metallic nanomaterials already used in a broad range of industrial applications. In the present study these two nanomaterials were characterized by Raman spectroscopy, transmission electron microscopy, zeta potential, and dynamic light scattering, and their biological properties were compared in terms of cytotoxicity, proliferation, and gene expression in human keratinocyte HaCaT cells. The results showed that both AgNPs and CNFs present similar time-dependent cytotoxicity (EC_50_ of 608.1 µg/mL for CNFs and 581.9 µg/mL for AgNPs at 24 h) and similar proliferative HaCaT cell activity. However, both nanomaterials showed very different results in the expression of thirteen genes (superoxide dismutase 1 (*SOD1*), catalase (*CAT)*, matrix metallopeptidase 1 (*MMP1)*, transforming growth factor beta 1 (*TGFB1)*, glutathione peroxidase 1 (*GPX1*), fibronectin 1 (*FN1*), hyaluronan synthase 2 (*HAS2*), laminin subunit beta 1 (*LAMB1*), lumican (*LUM*), cadherin 1 *CDH1,* collagen type IV alpha (*COL4A1*), fibrillin (*FBN*), and versican (*VCAN*)) treated with the lowest non-cytotoxic concentrations in the HaCaT cells after 24 h. The AgNPs were capable of up-regulating only two genes (*SOD1* and *MMP1*) while the CNFs were very effective in up-regulating eight genes (*FN1, MMP1, CAT, CDH1, COL4A1, FBN, GPX1,* and *TGFB1*) involved in the defense mechanisms against oxidative stress and maintaining and repairing tissues by regulating cell adhesion, migration, proliferation, differentiation, growth, morphogenesis, and tissue development. These results demonstrate CNF nanomaterials’ unique great potential in biomedical applications such as tissue engineering and wound healing.

## 1. Introduction

Nanotechnology is an emerging field of functional materials on the scale of nanometers at least in one dimension with a broad range of advanced applications such as medical imaging and nanomedicine [1,2,3,4]. Carbon nanofibers (CNFs) are one-dimensional, highly hydrophobic and non-polar filamentous hollow carbon-based nanomaterials (CBNs) that are cost-effective, have good electrical, thermal and mechanical properties [5,6], and show great promise in biomedical applications [7,8]. CNFs can be used to produce conductive composites [9] for biomedical approaches that require electrical stimulation [10] and are produced at a lower cost and higher purity than other CBNs such as carbon nanotubes (CNTs) [11].

While carbon nanostructures in the form of multiwalled carbon nanotubes (MWCNTs), CNFs, and carbon nanoparticles have shown size-dependent cytotoxicity in vitro in lung tumor cells [12], cytotoxicity tests have revealed a concentration- and time-dependent loss of lung fibroblasts, showing that CNFs are less dangerous than single-walled carbon nanotubes (SWCNTs) [13]. CNFs with diameters of 10 μm and 100 nm did not show toxicological activity in mouse keratinocytes (HEL-30), in contrast with 10 nm diameter MWCNTs and 1 nm diameter SWCNTs, which reduced cell viability in a time-dependent manner up to 48 h [14]. CNFs have also shown potent antibacterial properties against the clinically-relevant multidrug-resistant bacteria methicillin-resistant *Staphylococcus epidermidis* [15] and have been used to enhance the antiviral properties of composite materials [8]. CNFs have been combined with biopolymers to produce non-cytotoxic composites with improved physical and biological properties [16,17,18] in terms of mechanical, thermal, wettability, cell adhesion, and proliferation properties. This type of CBN possesses photocatalytic properties that can enhance its antibacterial properties when it is irradiated with light-emitting diodes [19].

Silver nanoparticles (AgNPs) have been studied in greater depth than CNFs. They are also cost-effective and possess excellent antimicrobial properties [20,21,22,23,24]. In fact, AgNPs are already broadly used in wound dressings for healing processes and treating burns in biomedicine as well as in the food and textile industries and in paints, household products, catheters, implants, and cosmetics and in combination with many types of materials to prevent infection [25,26,27,28,29,30]. These nanoparticles have great potential for use in dermatology and wound healing because of their prolonged capacity to release silver ions showing a concentration-dependent toxic effect in HaCaT cells [31]. Topical delivery of AgNPs promotes wound healing because they exert positive effects due to their antimicrobial activity, reduction action in wound inflammation, and the modulation effect of fibrogenic cytokines [32]. Varying AgNP morphologies have been reported to have different toxic effects against microorganisms, HaCaT keratinocytes, and to affect skin deposition [33]. Their chemopreventive efficacy has been demonstrated in HaCaT cells with a significant reduction in cyclobutene-pyrimidine-dimer formation after DNA damage induced by UVB irradiation [34], which provides great potential for preventing skin carcinogenesis. AgNPs’ UVB-protective efficacy in human keratinocytes depends on their size [35]. Thus, pre-treating HaCaT cells with small AgNPs (10–40 nm) was effective in protecting skin cells from UVB-radiation-induced DNA damage and from UV-radiation-induced apoptosis. However, no protection was obtained by using 60 and 100 nm AgNPs.

AgNPs are being increasingly used in the healthcare sector and consumer products and many commercial products now contain these nanoparticles for topical application to human skin. However, despite their growing number of applications comprehensive biological information still needs further research because of the many controversial results published on their safety [29]. For example, AgNPs showed reduced cell viability and metabolism as well as proliferative and migratory potential of primary normal human epidermal keratinocytes (NHEKs) at different concentrations [36]. NHEKs have been shown to be more susceptible to the application of AgNPs than normal human dermal fibroblasts (NHDFs). A comparative study was made of the effects of AgNPs and ionic silver (Ag^−1^) in terms of cell viability, inflammatory response, and DNA damage in normal NHDFs and NHEKs [37]. This study showed that Ag^−1^ is more toxic than AgNPs in both NHDFs and NHEKs. However, microorganisms are known to be capable of developing resistance mechanisms against silver [38,39] and the current excessive use of AgNPs as antibacterial compounds in many areas is increasing its potential risk to humans and the environment [40].

In this regard, alternative broad-spectrum antimicrobial carbon-based nanomaterials such as CNFs are characterized by their low risk of inducing microbial resistance [41], which shows their promise in providing long-lasting solutions in biomedicine. In the present study we analyzed the effects of AgNPs and CNFs on human epidermal HaCaT keratinocyte cells in terms of time-dependent cytotoxicity and their possible biomedical applications when used at low non-cytotoxic concentrations as proliferative agents. We also analyzed their capacity to modify the gene expression of the thirteen genes (superoxide dismutase 1 (*SOD1*), catalase (*CAT*), matrix metallopeptidase 1 (*MMP1*), transforming growth factor beta 1 (*TGFB1*), glutathione peroxidase 1 (*GPX1*), fibronectin 1 (*FN1*), hyaluronan synthase 2 (*HAS2*), laminin subunit beta 1 (*LAMB1*), lumican (*LUM*), cadherin 1 *CDH1,* collagen type IV alpha (*COL4A1*), fibrillin (*FBN*), and versican (*VCAN*)) associated with oxidative stress, the extracellular matrix, and protein synthesis for the maintenance and repair of different tissues. The expression of these genes is of interest for biomedical applications such as tissue engineering and wound healing.

## 2. Materials and Methods

### 2.1. Materials

Silver nanopowder (<150 nm particle size, product code 484059, 99% trace metals basis) was purchased from Sigma-Aldrich (Zwijndrecht, Switzerland). Carbon nanofibers (CNFs) were provided by Graphenano (Yecla, Spain). These CNFs were previously characterized by high-performance electron microscopy with elemental analysis (EDS) which showed that they were irregular one-dimensional hollow filaments with a wide range of diameters (22.7 ± 11.9 nm) and lengths (737.8 ± 522.4 nm) and the expected carbon to oxygen atom ratio (C/O ratio of 37.4) [5]. Fetal bovine serum (FBS), DMEM low glucose, penicillin–streptomycin (P/S), L-glutamine and epidermal growth factor (EGF) were obtained from Life Technologies (Gibco, Karlsruhe, Germany). An RNA purification kit was obtained from Norgen Biotek Corp (Ontario, Canada), and a PrimeScript™ RT Reagent Kit (Perfect Real Time) from Takara Bio Inc (Otsu, Japan).

### 2.2. Material Characterization

Transmission electron microscopy images were obtained by a JEOL 2100 electron microscope with a LaB_6_ thermoionic gun at 200 kV. The samples were dispersed in aqueous solution in ultrasound and dropped onto a (grid type) sample holder composed of Cu and C. Raman spectroscopy was performed by an NRS-3100 spectrometer (JASCO) coupled to a silicon CCD detector and an argon-ion laser (Melles Griot, 514.5 nm, 200 mW). The zeta potential (ζ), dynamic light scattering (DLS), and polydispersity index (PdI) values were obtained from a Zetasizer NanoZS (Malvern, UK). The ζ values were obtained in deionized water (pH = 3, 5, 7, 10, and 12) with pH variation using HNO_3_ (Synth, 70%) and NH_4_OH (Synth, 24%). The DLS technique was used to evaluate the particle hydrodynamic size of the two materials in water and in the Dulbecco’s modified Eagle medium (DMEM) used for the biological characterization.

### 2.3. Culture Maintenance

Immortalized human keratinocyte cell line HaCaT were cultured in DMEM low glucose, supplemented with FBS 10%, L-glutamine 2%, and P/S 1% in a humidified atmosphere at 5% CO_2_ and 37 °C. Cell medium was changed three times per week, and cells were trypsinized and resuspended in medium at low density when the culture reached 80% of confluence.

### 2.4. Preparation of Nanomaterial Stock Solutions

Nanomaterial stock solutions were prepared in sterile DMEM low glucose supplemented with P/S and L-glutamine, but not FBS, and were sonicated for 2 h to obtain a completely homogeneous solution of the different compounds in the medium. A medium vial was exposed to the same conditions to be used not only as the control group but also for the subsequent dilutions of the nanomaterials. The stocks solutions were used immediately after sonication.

### 2.5. Cytotoxicity Assay

Human keratinocytes were seeded onto 96-well plates at a density of 1 × 10^4^ cells/well and grown in an incubator with a humidified atmosphere (5% CO_2_ and 37 °C). After 24 h the medium was changed with 100 µL with the corresponding concentrations, ranging from 0 to 800 µg/mL of each compound. The compounds and concentration batteries were tested per sextuplicate for 3, 12, and 24 h to evaluate different cytotoxicity endpoints at different times. Six replicate samples of each concentrations were measured plus an untreated control group (also without FBS). The concentrations selected to calculate the EC_50_ of both compounds were 20, 40, 80, 150, 300, 500, and 800 µg/mL at 12 and 24 h. Cytotoxicity was evaluated by the 3-(4, 5-dimethylthiazol-2-yl)-2, 5-diphenyl tetrazolium (MTT) assay. Cells with MTT reagent were incubated for 5 h in the same conditions; formazan crystals were then solubilized with DMSO and cell viability was calculated from the absorbance values at 550 nm measured in a Varioskan micro plate reader (ThermoScientific, Canada). The same experiment was carried out in parallel to remove false positives due to cell pigmentation with AgNPs and CNFs, excluding the MTT reagent so that the background color could be subtracted from the final absorbance values.

### 2.6. Proliferation Assay

As a safeguard against cytotoxicity by increasing time exposure, two non-toxic ten-time diluted concentrations were selected according to the cytotoxicity results at 24 h. Cells were seeded in a 96-well culture plate at a density of 5 × 10^3^ cells/well. The stock solution was prepared following the same procedure as indicated in Materials and Methods but with FBS 0.5% instead of 0%. Cells were cultured for 72 or 96 h in a humidified atmosphere (5% CO_2_ and 37 °C). A proliferative positive control was included and treated with epidermal growth factor (EGF) at 15 ng/mL. Cell proliferation was measured by the MTT assay as conducted in the cytotoxicity assay. A sextuplicate was run for the different conditions and exposure periods.

### 2.7. Gene Expression

Gene expression analysis was performed in triplicate using two non-toxic concentrations based on the cytotoxicity results performed at 24 h. Cells were seeded in a 6-well culture plate at a density of 1.5 × 10^6^ cells/well. After incubation for 24 h with the different nanomaterials, the supernatant was aspirated and the cells were washed twice with PBS 1× for RNA extraction, cDNA synthesis, and RT-qPCR. Data were analyzed by QuantStudio^TM^ Design & Analysis Software (ThermoFisher, Canada). The primers for target genes (Table A1 in Appendix A) and reference gene (β-actin/ACTB) were obtained on Primer-Blast software [42]. Data normalization was based on the expression of the reference gene.

### 2.8. Statistical Analysis

The statistical analysis was performed by ANOVA followed by multiple Tukey’s post-hoc analysis. Probit analysis was used to determine the median effective concentration (EC_50_) values. GraphPad Prism 6 software was used in the statistical analysis at a significance level of at least *p* < 0.05.

## 3. Results

### 3.1. Material Characterization

**Figure 1:**
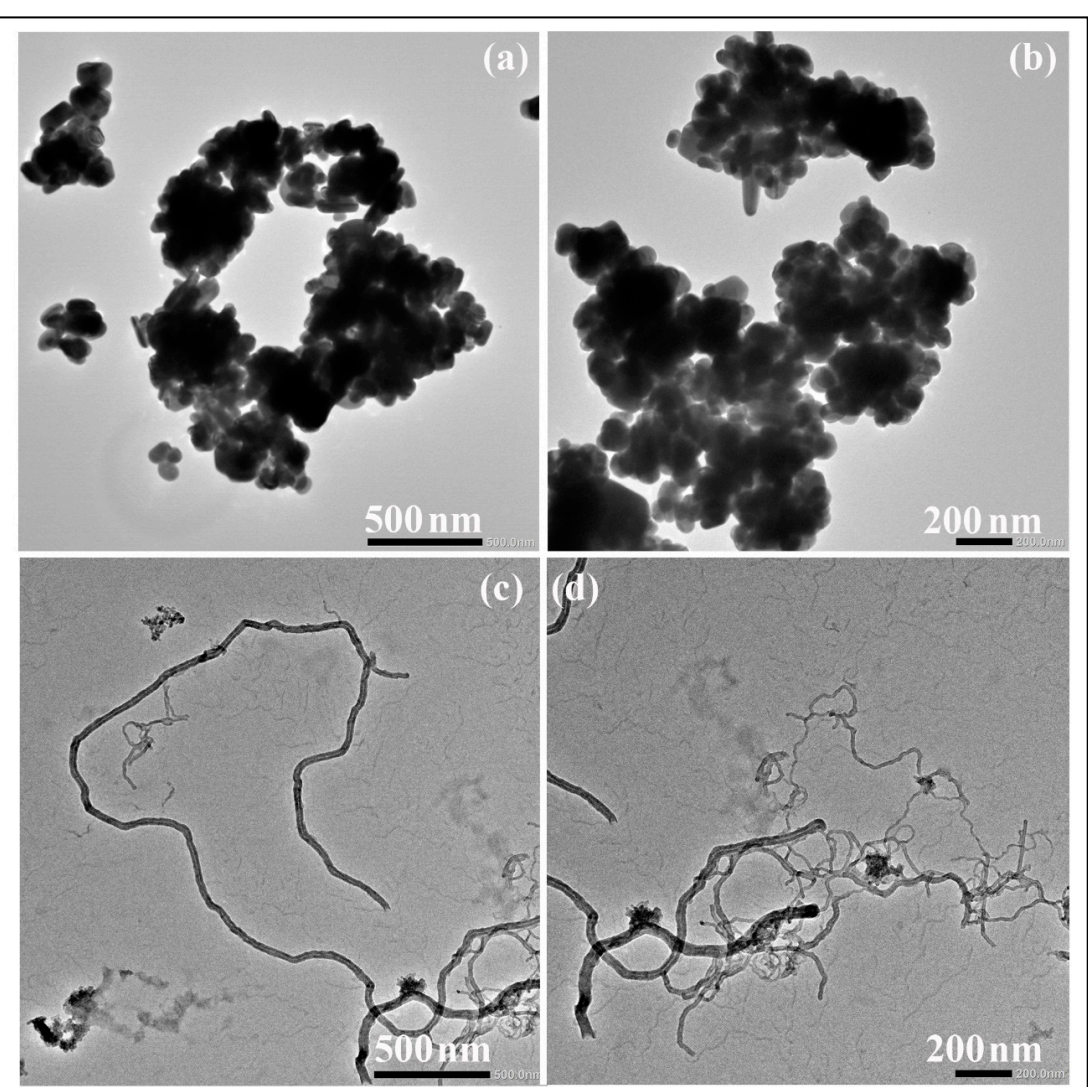
Transmission electron micrographs of silver nanoparticles (**a**,**b**) and carbon nanofibers (**c**,**d**) at two different magnifications.

The AgNPs and CNFs were characterized by transmission electron microscopy, Raman spectroscopy, zeta potential, and DSL. The morphologies of the AgNPs and CNFs used are shown in Figure 1 at two magnifications.

The DLS technique performed to evaluate the particle hydrodynamic size of the two nanomaterials showed larger size values (1693 and 1142 nm) in the DMEM used for the biological characterization than in water (461.3 and 811.2 nm) for both AgNPs and CNFs (Table 1).

**Figure 2:**
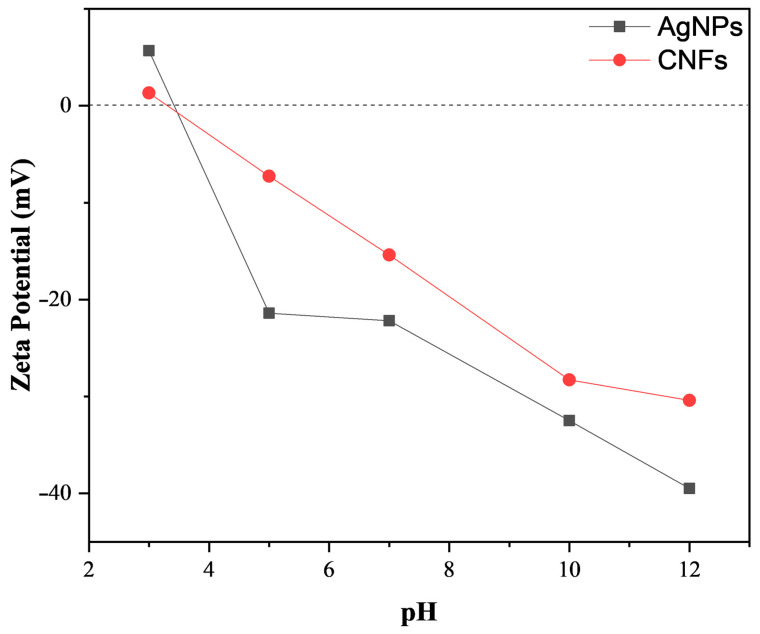
Zeta potential (ζ) in mV of silver nanoparticles (AgNps) and carbon nanofibers (CNFs) as a function of pH in a water solution.

The two types of nanomaterial showed different zeta potential (ζ) as a function of pH in water solution (Figure 2).

**Figure 3:**
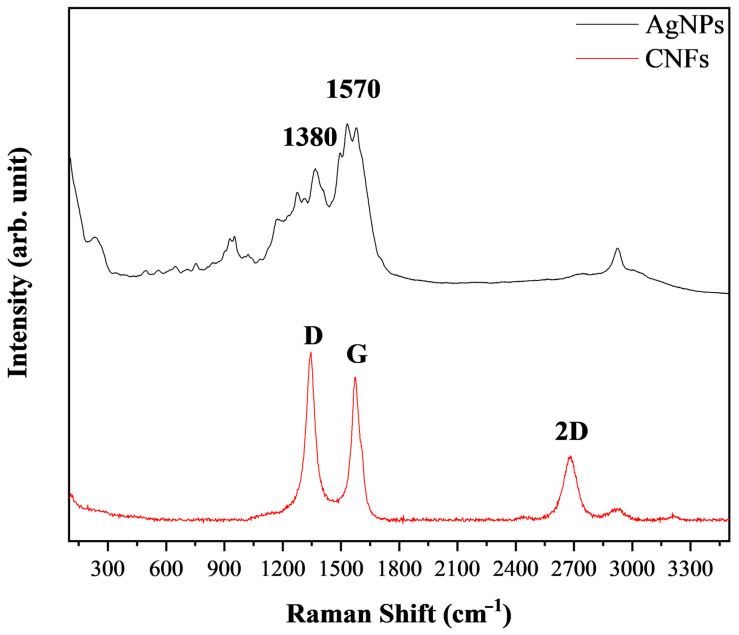
Rama spectra of silver nanoparticles, AgNPs and carbon nanofibers, CNFs.

The Raman spectra of the two chemically different nanomaterials (metallic nanomaterial versus carbon-based nanomaterial) are shown in Figure 3.

The most representative AgNP Raman peaks (1380 and 1570 cm^−1^) and CNFs (D, G, and 2D bands) are indicated in Figure 3.

### 3.2. Biological Properties

The AgNPs’ and CNFs’ biological properties in terms of time-dependent cytotoxicity, proliferation, and gene expression in human keratinocytes cells are described in the following subsections.

### 3.3. Cytotoxicity Assay

**Figure 4:**
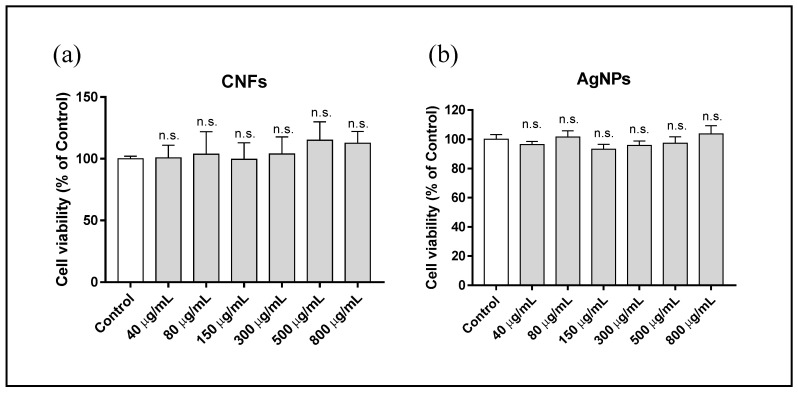
Cytotoxicity assay in human keratinocyte (HaCaT) cells after 3 h exposure to AgNPs (**a**) or CNFs (**b**) at different concentrations ranging from 0 (control) to 800 µg/mL. Cytotoxicity was evaluated by MTT assay. The results are given as a % of the control group. Data are shown as the mean ± standard deviation of six replicates. The ANOVA results of the different AgNP or CNF concentrations with respect to the control are indicated in the plot; n.s: not significant.

A study was made of the cytotoxicity of different concentrations ranging from 0 (control) to 800 µg/mL of AgNPs and CNFs in HaCaT cells for different exposure times (3, 12, and 24 h). The results showed that none of the AgNP and CNF concentrations was cytotoxic for the HaCaT cell line at 3 h of exposure (Figure 4).

**Figure 5:**
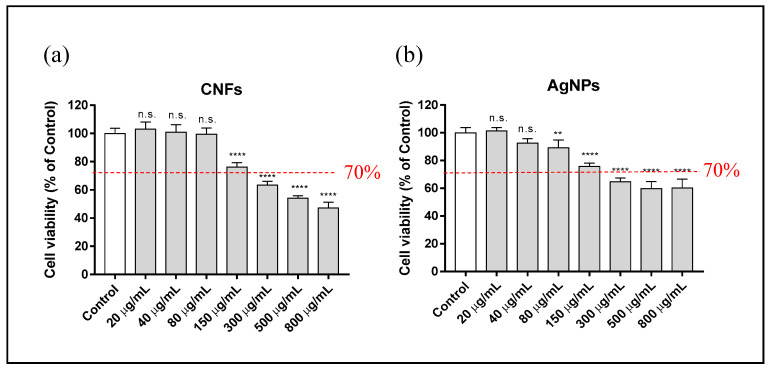
Cytotoxicity assay in human keratinocyte (HaCaT) cells after 12 h exposure to AgNPs (**a**) or CNFs (**b**) at different concentrations ranging from 0 (control) to 800 µg/mL. Cytotoxicity was evaluated by the MTT assay. Results are represented as a % of the control group. Data are shown as the mean ± standard deviation of six replicates. The ANOVA results of the different AgNP or CNF concentrations with respect to control are indicated in the plot. ** *p* < 0.01; **** *p* < 0.0001; n.s: not significant. The cell viability limit (70%) for the compounds to be considered non-cytotoxic is indicated.

However, the longer time exposure of up to 12 h required less AgNP and CNF exposure concentration than the concentrations used for 3 h to avoid cytotoxic effects (Figure 5).

**Figure 6:**
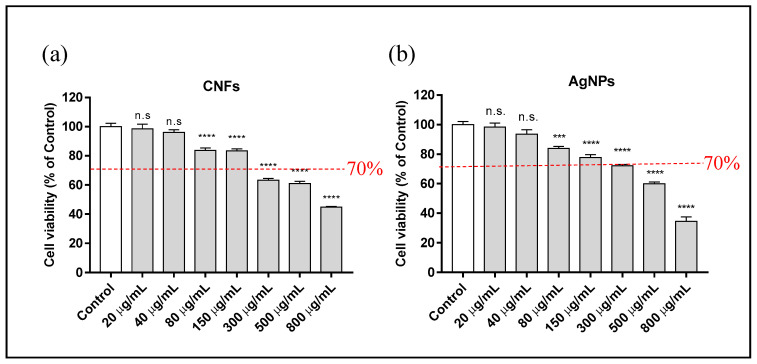
Cytotoxicity assay in human keratinocyte (HaCaT) cells, after 24 h exposure to AgNPs (**a**) or CNFs (**b**) at different concentrations ranging from 0 (control) to 800 µg/mL. Cytotoxicity was evaluated by the MTT assay. Results were represented as a % of the control group. Data are given as the mean ± standard deviation of six replicates. The ANOVA results of the different AgNP or CNF concentrations with respect to control are indicated in the plot. *** *p* < 0.001; **** *p* < 0.0001; n.s: not significant. The limit of cell viability (70%) for the compounds to be considered non-cytotoxic is indicated.

The results show a negative correlation with cell viability, indicating that the toxicity of these compounds is dose-dependent. The cytotoxicity assay results at 24 h of exposure time in HaCaT cells also showed a non-cytotoxic concentration of ≤150 µg/mL for CNFs and AgNPs (Figure 6).

From these results at 24 h of exposure, the mean effective concentrations (EC_50_) were determined for AgNPs and CNFs (Table 2).

### 3.4. Proliferation Assay

**Figure 7:**
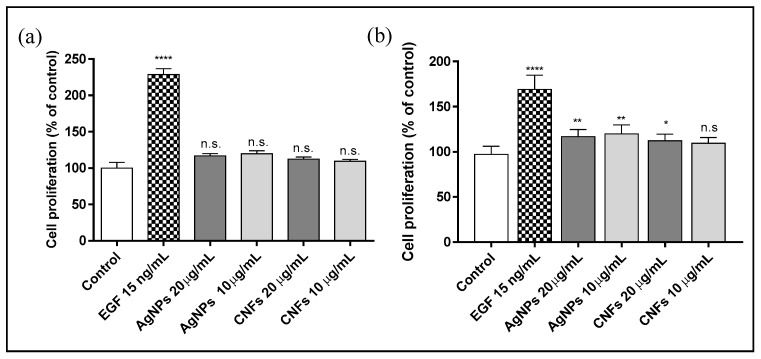
Proliferative activity of AgNPs or CNFs in human keratinocyte (HaCaT) cells stimulated by their exposure to non-cytotoxic concentrations (10 and 20 µg/mL) for 72 (**a**) or 96 (**b**) hours. Data are shown as the mean ± standard deviation of six replicates. The ANOVA results of the AgNP and CNF concentrations and epidermal growth factor (EGF) with respect to control are given in the plot. * *p* < 0.05; ** *p* < 0.01; **** *p* < 0.0001; n.s: not significant.

The proliferative activity of AgNPs and CNFs in the keratinocytes cell line was studied using two non-cytotoxic concentrations (10 and 20 μg/mL) based on the previous results obtained from the cytotoxic assay at 24 h (Figure 6) to avoid toxic effects by increasing exposure time to 72 and 96 h (Figure 7).

The results showed that 72 h was not long enough to induce cell proliferation. However, AgNPs at both concentrations (20 and 10 µg/mL) and CNFs, only at 20 µg/mL, showed a statistically significant increase in cell growth.

### 3.5. Gene Expression

**Figure 8:**
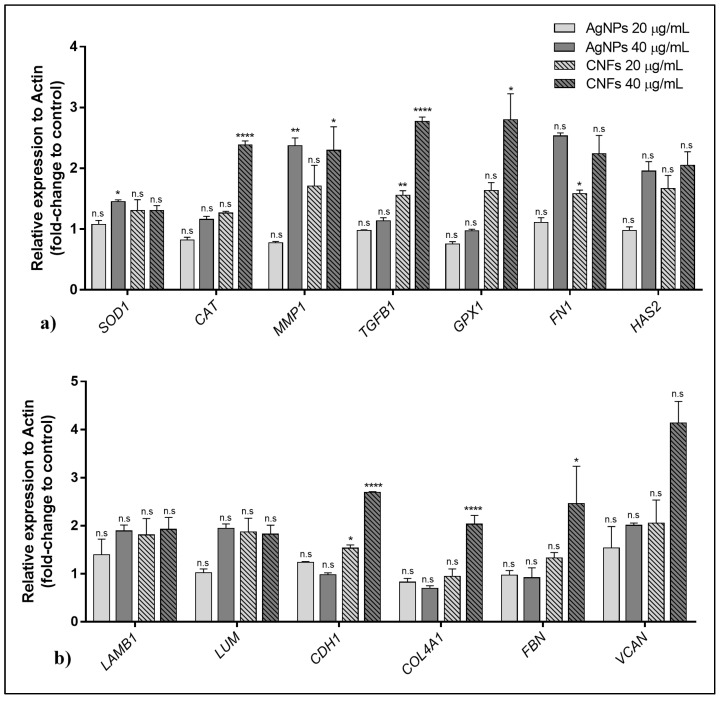
Effect of AgNPs and CNFs on the expression of thirteen genes: (**a**) *SOD1, CAT, MMP1, TGFB1, GPX1, FN1,* and *HAS2* and (**b**) *LAMB1, LUM, CDH1, COL4A1, FBN,* and *VCAN* at two non-cytotoxic concentrations (20 and 40 µg/mL) in human keratinocyte (HaCaT) cells after 24 h. Data are shown as mean ± standard deviation from three replicates. The results are given as fold-change of control and relative expression to ACTB. * *p* < 0.05; ** *p* < 0.01; **** *p* < 0.0001; n.s: not significant).

The effect of AgNPs and CNFs on the expression of thirteen genes are shown in Figure 8a (genes SOD1, CAT, MMP1, TGFB1, GPX1, FN1 and *HAS2*) and Figure 8b (genes *LAMB1, LUM, CDH1, COL4A1, FBN* and *VCAN*) at two non-cytotoxic concentrations (20 and 40 µg/mL) in human keratinocyte cells after 24 h.

These results show that exposure to CNFs at 40 µg/mL produces gene overexpression in most of the studied genes (*CAT, MMP1, TGFB1, GPX1, CDH1, COL4A1*, and *FBN*), while AgNPs were only able to induce expression changes in two genes (*SOD1* and *MMP1*).

## 4. Discussion

The TEM images show that these two nanomaterials present very different morphologies (Figure 1). CNFs are filamentous materials of micrometric length and an average nanometric diameter of 21.73 ± 9.59 nm, and their morphology is apparently similar to that of carbon nanotubes (CNTs). However, unlike CNTs, CNFs present disordered graphitic layers [17]. These AgNPs present smaller sizes that vary in form from spherical to ellipsoidal. AgNPs have an average individual particle size of 110.10 ± 33.85 nm, but they are presented in the form of several agglomerates. According to the TEM images, the DLS results (Table 1) show larger size values than individual particles for both CNFs and AgNPs. As expected, the particle size values depend on whether the nanofluid is prepared with water solution or DMEM [43]. The PdI values of the samples measured by this technique are also shown in Table 1 to provide a particle aggregation parameter. Both AgNP and CNF show PdI values slightly higher in DMEM than in water, which could be attributed to the higher CNF and AgNP aggregation in this medium and could explain their greater DLS size with respect to that measured in water. However, PdI increased more for AgNPs than for CNFs, in good agreement with the greater DSL size increase found for AgNPs in DMEM. These large differences of particle aggregation in DMEM must be related to the very different morphology of each type of nanomaterial and their different surface charge or zeta potential (ζ) shown in Figure 2.

Raman spectroscopy provides valuable nanostructural information on CBNs and metal nanoparticles [44,45,46]. The Raman spectrum of the CNFs showed the three typical bands (D, G, and 2D) at ~1350, 1595, and 2690 cm^−1^, respectively, and an I_D_/I_G_ ratio of 1.17, typical of this type of CBN, which presents a higher degree of disorder than CNTs (Figure 3) [47]. The AgNP Rama spectrum showed two main vibrational modes with the maxima at about 1380 and 1570 cm^−1^, as expected [46].

CNF and AgNP cytotoxicity was studied at concentrations ranging from 0 (control) to 800 µg/mL in HaCaT cells for different exposure times (3, 12, and 24 h). None of the concentrations of both nanomaterials was cytotoxic for the HaCaT cell line at 3 h of exposure (see Figure 4).

As expected, both CNFs and AgNPs showed a negative correlation with cell viability, indicating a dose-dependent toxicity of these compounds (Figure 5). Considering a limit of reduction to 70% of cell viability with respect to the control, both compounds showed similar cytotoxicity in human keratinocyte HaCaT cells with a non-cytotoxic concentration of ≤150 µg/mL after 12 h of exposure. The cytotoxicity assay at 24 h of exposure time also showed similar results for both compounds (see Figure 6), also with a non-cytotoxic concentration of ≤150 µg/mL. CNFs have shown higher cytotoxicity than single-wall carbon nanotubes [13]. However, in this study CNFs were shown to be much less cytotoxic in human keratinocytes than multi-layer graphene oxide [48]. The mean effective concentrations (EC_50_) of AgNPs and CNFs at 24 h of exposure also show similar values in Table 2. It is important to emphasize that CNFs are CBNs had much lower toxicity than other CBNs such as multi-layer graphene oxide (EC_50_ of 4.087 μg/mL at 24 h) and few-layer graphene oxide (EC_50_ of 62.8 μg/mL at 24 h) in human keratinocyte HaCaT cells. Our cytotoxicity results for the AgNPs with spherical to ellipsoidal shapes are in good agreement with previous results on AgNPs in the form of plates (z-potential −37.5 mV) and spheres (z-potential −30.4 mV) in human HaCaT keratinocytes in vitro, which showed an IC_50_ of 78.65 μg/mL (95% CI 63.88, 96.83) and 1004 µg/mL (95% CI 286.8, 3516) at 24 h, respectively [33]. AgNPs are more promising than silver cations because they are less cytotoxic (e.g., the IC_50_ of silver nitrate is 7.85 μg/mL (95% CI 1.49, 14.69)).

Both nanocompounds showed a slightly statistically significant proliferative activity at 96 h of exposure time. A shorter exposure time (72 h) was not long enough to induce cell proliferation, as has been found for other nanomaterials such as GO [48]. CNFs at 10 μg/mL was not a high enough concentration to induce any proliferative effect.

The effect of the two different nanomaterials on the expression of thirteen genes (Table A1 in Appendix A) involved in the activation or inhibition of different metabolic routes such as oxidative stress, extracellular matrix, and synthesis of proteins related to the maintenance and repair of different tissues was analyzed in human keratinocytes cells. CNFs were able to up-regulate eight genes (*CAT, MMP1, TGFB1, GPX1, FN1, CDH1, COL4A1*, and *FBN*)—four genes (*MMP1, TGFB1, FN1*, and *CDH1*) at a concentration of 20 μg/mL and seven genes (*CAT, MMP1, TGFB1, GPX1, CDH1, COL4A1,* and FBN) at 40 μg/mL. Exposure of HaCaT cells to CNFs increased the expression of FN1, which regulates cell adhesion and migration [49], and *TGFB1*, involved in cell proliferation, differentiation, and growth [50,51]. These results are in agreement with those reported previously on the enhancement of proliferative activity and cell adhesion of canine adipose-derived mesenchymal stem cells with the addition of CNFs in poly(3-hydroxybutyrate-co-3-hydroxyvalerate) [18]. The expression of catalase (*CAT*) and glutathione per-oxidase 1 (*GPX1*) genes that encode the synthesis of enzymes involved in the neutralization of hydrogen peroxide with an antioxidant effect, was also up-regulated in HaCaT cells after 24 h of exposure to CNFs. The activation of these two genes has been reported to be associated with defense mechanisms against stressors in human skin cells during photoaging as protective oxidative activity against UVA radiation [52,53,54,55]. The CNFs also increased the expression of the genes involved in the synthesis of glycoproteins such as cadherin 1 (*CDH1*) and fibrillin (*FBN*), which are essential in the morphogenesis and development of normal tissue by connecting cells with each other [56,57]. The *COL4A1* gene, which is abundant in the dermis, and the *MMP1* gene, which is involved in the breakdown of the extracellular matrix in normal physiological processes, were also up-regulated after exposure of keratinocytes to CNFs for 24 h. The up-regulation of four of these eight genes (*FN1, TGFB1, CAT* and *CDH1*) has also been observed in other CBNs such as multilayer GO [48]. However, the increase of the expression of the *GPX1* gene was not observed in multilayer GO, probably due to the low non-cytotoxic concentration used (0.05 μg/mL) in that study because, as was found in the present study, this increase was only found at a much higher CNF concentration (40 μg/mL). Only one (*MMP1*) out of these eight genes was up-regulated by exposing HaCaT cells to AgNPs and only using the highest non-cytotoxic concentration (40 μg/mL). Nonetheless, AgNPs were able to increase the expression of *SOD1*, which encodes an isozyme that destroys free superoxide radicals in the body by binding copper and zinc ions [58].

AgNP toxicity has also been evaluated in NIH 3T3 mouse embryo fibroblasts and showed cell damage via the generation of ROS [26]. ROS may contribute to tissue damage and participate in cellular events such as signal transduction, proliferative response, and protein redox regulation and can modulate the expression of numerous genes [26,59,60]. A study of the cytotoxic mechanisms of AgNPs in keratinocytes showed them to be related to oxidative damage and inflammation, as shown by increased concentrations of reactive oxygen species (ROS), malondialdehyde (MDA), interleukin-1 alpha, interleukin-6, and interleukin-8 [61]. The effect of AgNPs on the metabolic profile of a human HaCaT epidermis keratinocyte line exposed for 48 h to 30 nm citrate-stabilized spherical AgNPs (10 and 40 μg/mL) by nuclear magnetic resonance metabolomics showed up-regulated glutathione-based antioxidant protection, increased glutaminolysis, down-regulated tricarboxylic acid (TCA) cycle activity, energy depletion, and cell membrane modification [62]. However, the damage produced by the AgNPs used in the current study was not similar to that produced by smaller AgNPs, since AgNP cytotoxicity is influenced by variations in size, shape, and surface electric charges [63]. Cellular uptake and generation of ROS was also found in the murine RAW264.7 macrophages after exposure to CNF, but not after exposure to SWCNT, another type of CBN [13]. CBN toxicity has been reported to depend on dimension and composition [14].

## 5. Conclusions

The results of this work can be summarized as follows: (i) AgNPs are smaller and present a very different morphology to filamentous CNF carbon-based materials; (ii) AgNPs had higher negative zeta potential (ζ), from pH 5–12, than CNFs and similar time-dependent cytotoxicity (EC_50_ of 608.1 µg/mL for CNFs and 581.9 µg/mL for AgNPs at 24 h); (iii) both nanomaterials showed similar proliferative activity at 20 μg/mL after 96 h in the HaCaT cells; (iv) this study provides the first comparison of time-dependent cytotoxicity, proliferation, and gene expression in human keratinocyte HaCaT cells between CNFs and AgNPs; (v) AgNPs were capable of up-regulating only two genes (*SOD1* and *MMP1*) out of the thirteen genes analyzed. However, CNFs were able to up-regulate eight genes (*FN1, MMP1, CAT, CDH1, COL4A1, FBN, GPX1*, and *TGFB1*), which possess many important properties required for biomedical applications, such as defense mechanisms against oxidative stress and tissue maintenance and repair. These results thus show great promise as they open up the possibility of using antimicrobial CNF nanomaterials in a broad range of biomedical applications, including tissue engineering and wound healing.

## Figures and Tables

**Table 1:** Particle hydrodynamic sizes in nm of the two materials in water solution and in the Dulbecco’s modified Eagle medium (DMEM) used for biological characterization by the dynamic light scattering technique. The polydispersity index (PdI) values of the two materials are also given.

Material	DLS (nm)		PdI	
	Water	DMEM	Water	DMEM
AgNPs	461.3	1693	0.471	0.533
CNFs	811.2	1142	0.586	0.615

**Table 2:** Mean effective concentration (EC_50_) of keratinocyte (HaCaT) cells after treatment with AgNPs and CNFs at an exposure of 24 h. Mean EC_50_ in µg/mL, confidence limits 95% (CI) and goodness of fit (R square) are shown.

Nanomaterial	EC_50_ (µg/mL)	95% CI	R Square
AgNPs	581.9	515.2–670.4	0.9037
CNFs	608.1	531.4–709.5	0.9308

## Data Availability

Data is contained within the article.

## Appendix A

**Table A1:** Gene symbol, gene name, oligo sequences, and function of specific genes used in RT-qPCR measurements.

Gene Symbol (Access Number)	Gene Name	Oligo Sequences	Function
*ACTB* (NM_001101)	Actin beta	5′-CCATGCCCACCATCACGC-3′	Highly conserved protein involved in cell motility, structure, and integrity
		5′-CACAGAGCCTCGCCTTTG-3′	
*CAT* (NM_001752)	Catalase	5′-TGAATGAGGAACAGAGGAAACG-3′	Encodes catalase, a key antioxidant enzyme in the body’s defense against oxidative stress
		5′-AGATCCGGACTGCACAAAG-3′	
*MMP1* (NM_001145938)	Matrix metallopeptidase 1	5′-GGACCATGCCATTGAGAAAG-3′	Involved in the breakdown of extracellular matrix in normal physiological processes
		5′-TCCTCCAGGTCCATCAAAAG-3′	
*GPX1* (NM_000581)	Glutathione peroxidase 1	5′-TTTGGGCATCAGGAGAACGC-3′	Catalyze the reduction of organic hydroperoxides and hydrogen peroxide by glutathione, and thereby protect cells against oxidative damage
		5′-ACCGTTCACCTCGCACTTC-3′	
*COL4A1* (NM_000088)	Collagen type I alpha 1	5′-CAAGGGCGACAGAGGTTTGC-3′	Abundant in bone, cornea, dermis, and tendon. Mutations in this gene are associated with osteogenesis imperfect types I-IV
		5′-AAAACTCACCAGGCTCCCCC-3′	
*TGFB1* (NM_000660)	Transforming growth factor beta 1	5′-AGCTGTACATTGACTTCCGCA-3′	Regulates cell proliferation, differentiation, and growth
		5′-TGTCCAGGCTCCAAATGTAGG-3′	
*HAS2* (NM_005328)	Hyaluronan synthase 2	5′-CCGAGAATGGCTGTACAATGC-3′	Serves a variety of functions, including space filling, lubrication of joints, and provision of a matrix through which cells can migrate
		5′-AGAGCTGGATTACTGTGGCAA-3′	
*LAMB1* (NM_002291)	Laminin subunit beta 1	5′-CAGGGTGTGCAGTCAGGGAA-3′	Implicated in a wide variety of biological processes including cell adhesion, differentiation, migration, signaling, neurite outgrowth, and metastasis
		5′-TGTGTCTGCGTTGAGGGTGT-3′	
*LUM* (NM_002345)	Lumican	5′-ACTTGGGTAGCTTTCAGGGCA-3′	Is the major keratan sulfate proteoglycan of the cornea but is also distributed in interstitial collagenous matrices throughout the body
		5′-TTCCTGGCATTGATTGGTGGT-3′	
*FN1* (NM_001306129)	Fibronectin 1	5′-GGCCAGTCCTACAACCAGT-3′	Involved in cell adhesion and migration processes including embryogenesis, wound healing, blood coagulation, host defense, and metastasis.
		5′-CGGGAATCTTCTCTGTCAGC-3′	
*VCAN* (NM_001126336)	Versican	5′-CTGGTCTCCGCTGTATCCTG-3′	Involved in cell adhesion, proliferation, migration, and angiogenesis and plays a central role in tissue morphogenesis and maintenance
		5′-ATCGCTGCAAAATGAACCCG-3′	
*CDH1* (NM_001317184)	Cadherin 1	5′-AACAGCACGTACACAGCCCT-3′	Loss of function of this gene is thought to contribute to cancer progression by increasing proliferation, invasion, and/or metastasis.
		5′-TCTGGTATGGGGGCGTTGTC-3′	
*FBN* (NM_000138)	Fibrillin 1	5′-ATCCAACCACGTGCATCAGT-3′	Extracellular matrix glycoprotein that serves as a structural component of calcium-binding microfibrils, providing force-bearing structural support in elastic and nonelastic connective tissue throughout the body
		5′-AGAGCGGGTATCAACACAGC-3′	
*SOD1* (NM_000454)	Superoxide dismutase 1	5′-GGTGTGGCCGATGTGTCT-3′	The protein encoded by this gene binds copper and zinc ions and is one of two isozymes responsible for destroying free superoxide radicals in the body
		5′-TCCACCTTTGCCCAAGTCA-3′	
